# Cerebellar pathology contributes to neurodevelopmental deficits in spinal muscular atrophy

**DOI:** 10.21203/rs.3.rs-6819992/v1

**Published:** 2025-06-11

**Authors:** Florian Gerstner, Sandra Wittig, Christian Menedo, Sayan Ruwald, Maria J Carlini, Adela Vankova, Leonie Sowoidnich, Gerardo Martín-López, Vanessa Dreilich, Andrea Alonso Collado, John G Pagiazitis, Oumayma Aousji, Chloe Grzyb, Amy Smith, Mu Yang, Francesco Roselli, George Z Mentis, Charlotte J Sumner, Livio Pellizzoni, Christian M Simon

**Affiliations:** 1 Carl-Ludwig-Institute for Physiology, Leipzig University, Leipzig, 04103, Germany; 2 Department of Neurology, Columbia University, New York, NY, 10032, USA; 3 Center for Motor Neuron Biology and Disease, Columbia University, New York, NY, 10032, USA; 4 Department of Neurology, Center for Biomedical Research (ZBF), Ulm University, Ulm, 89081, Germany; 5 German Center for Neurodegenerative Diseases (DZNE), Ulm, 89081, Germany; 6 Department of Neurology, Johns Hopkins University School of Medicine, Baltimore, MD, 21205, USA; 7 Department of Neuroscience, Johns Hopkins University School of Medicine, Baltimore, MD, 21205, USA; 8 Department of Genetic Medicine, Johns Hopkins University School of Medicine, Baltimore, MD 21218, USA; 9 Mouse Neurobehavioral Core Facility, Columbia University, New York, NY, 10032, USA; 10 Department of Pathology and Cell Biology, Columbia University, New York, NY, 10032, USA

## Abstract

Spinal muscular atrophy (SMA) is a neuromuscular disease characterized by ubiquitous SMN deficiency and loss of motor neurons. The persistence of motor and communication impairments, together with emerging cognitive and social deficits in severe Type I SMA patients treated early with SMN-restoring therapies, suggests a broader dysfunction involving neural circuits of the brain. To explore the potential supraspinal contributions to these emerging phenotypes, we investigated the cerebellum, a brain region critical for both motor and cognitive behaviors. Here, we identify cerebellar pathology in both *post-mortem* tissue from Type I SMA patients and a severe mouse model, which is characterized by lobule-specific Purkinje cell (PC) death driven by cell-autonomous, non-apoptotic p53-dependent mechanisms. Loss and dysfunction of excitatory parallel fiber synapses onto PC further contribute to cerebellar circuit disruption and altered PC firing. Furthermore, we identified impaired ultrasonic vocalization (USV) in a severe SMA mouse model—a proxy for early-developing social communication skills that depend on cerebellar function. Cell-specific rescue experiments demonstrate that intrinsic cerebellar pathology contributes to motor and social communication impairments independently of spinal motor circuit abnormalities. Together, these findings identify a pathogenic role for cerebellar dysfunction and broaden the understanding of SMA as a multi-regional neurodevelopmental disorder.

## Introduction

Neuronal degeneration and circuit dysfunction are central features of neurodegenerative diseases, driving progressive impairments in movement and cognition among other fundamental behaviors. While selective vulnerability of specific neuronal populations often defines clinical presentation, mounting evidence suggests that circuit-level disruption across interconnected brain regions contributes to disease progression and phenotypic complexity^1,2^. This is particularly relevant in motor neuron diseases that have been historically viewed as disorders solely driven by the loss of upper or lower motor neurons^3,4^. Clinically relevant behaviors such as locomotion, posture, and vocalization depend on distributed circuits spanning the spinal cord and the brain, raising the possibility that broader neuronal involvement shapes clinical outcomes^5–8^. However, our current understanding of the full spectrum of neural circuit deficits implicated in the etiology of motor neuron diseases is incomplete.

Spinal muscular atrophy (SMA) is a devastating neurodegenerative disease caused by a ubiquitous deficiency in the survival motor neuron (SMN) protein^8^. Traditionally defined by degeneration of spinal and bulbar motor neurons, the most common and severe form of SMA (Type I) leads to paralysis, speech impairment, and early death if left untreated^9–12^. SMN-restoring therapies have markedly improved survival but, even with early intervention, severe Type I SMA patients often exhibit persistent motor impairments alongside emerging social communication and cognitive deficits—including intellectual disability, absence of speech, and autism spectrum disorder (ASD)^10,13–17^. These clinical findings suggest that SMA likely affects broader neuronal circuits beyond spinal motor neuron networks.

Preclinical studies in animal models have been instrumental in identifying neuronal populations beyond motor neurons that contribute to SMA pathogenesis. In severe mouse models of the disease, preventing cell-autonomous p53-mediated death of motor neurons only partially rescues motor function^18–27^, supporting clinical observations that additional neuronal circuits contribute to the SMA phenotype. Accordingly, several studies have demonstrated that local spinal interneurons, proprioceptive sensory-motor circuits, and brainstem networks independently contribute to distinct aspects of motor dysfunction in mouse and fly models of SMA^21,22,25,28–33^. The clinical relevance of proprioceptive sensory-motor circuit pathology in the spinal cord has recently been confirmed in SMA patients^34,35^. However, the presence of communication and cognitive deficits in SMA patients suggests involvement of brain regions beyond the spinal cord, although the specific regions and underlying pathogenic mechanisms remain poorly defined.

Among candidate regions, the cerebellum is particularly compelling due to its central role in motor control, sensory-motor integration, cognition, social processing, and its known involvement in the pathogenesis of ASD, an emerging comorbidity in SMA^13,15,36–41^. In agreement, a number of SMA studies have reported cerebellar abnormalities^42–47^. However, the prevalence, underlying mechanisms, and functional significance of cerebellar pathology remain poorly understood.

Here, we identify a selective, cerebellar-autonomous pathology restricted to specific lobules associated with motor and social communication behaviors in a severe mouse model of SMA. Cerebellar pathology comprises progressive p53-dependent death of select pools of Purkinje cells (PCs) as well as cerebellar circuit dysfunction which includes disruption of both excitatory and inhibitory synaptic inputs onto PCs leading to their abnormal firing. We show that these changes contribute to motor deficits and to a newly identified phenotypic impairment in ultrasonic vocalization (USV). Furthermore, analysis of *post-mortem* tissue from Type I SMA patients confirmed both induction of p53 and loss of PCs in the cerebellum, strengthening the clinical relevance of our findings for human pathology. Together, our results identify cerebellar pathology as a key contributor to the neurodevelopmental disruption of motor and communication behaviors in SMA.

## Results

### PCs degenerate in a severe SMA mouse model and type I patients

**Figure 1:**
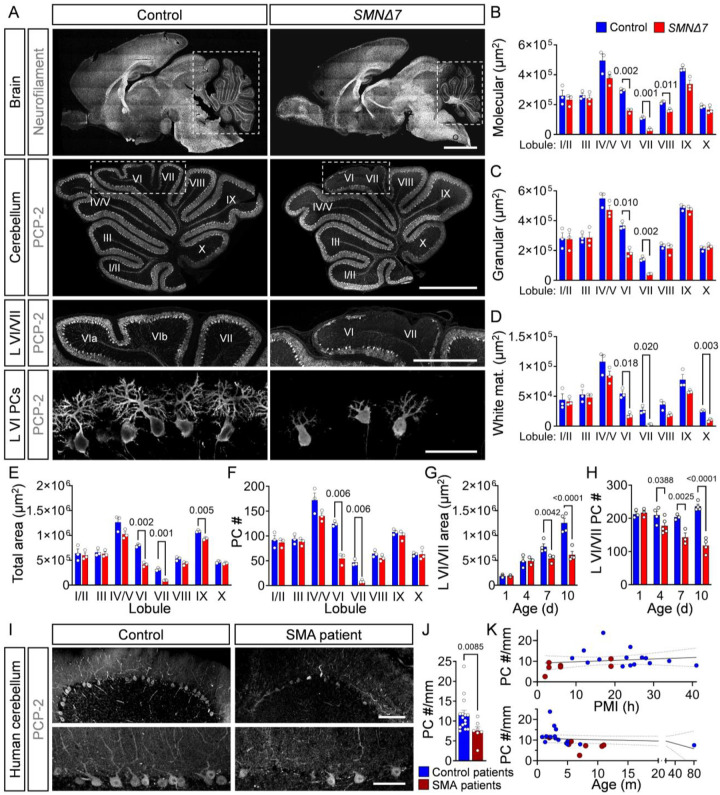
PC degeneration in a severe SMA mouse model and Type I patients. (**A**) Immunostaining of a section of the brain with antibodies against neurofilament M (first panel) and the cerebellum (second panel), cerebellar lobules VI/VII (third panel and lobule VI PCs (forth panel) with anti-PCP-2 antibodies from P10 control and *SMNΔ7* mice (scale bars: first panel = 2 mm, second panel = 1 mm, third panel = 0.5 mm, bottom panel = 50μm). Lobule-specific area quantification of the (**B**) molecular layer, (**C**) granular layer, (**D**) white matter, (**E**) total area, and (**F**) PC numbers in P10 control (N = 3) and *SMNΔ7* mice (N = 3). Time course analysis of the area (**G**) and PC numbers (**H**) of lobules VI/VII at P1, P4, P7, and P10 in control (N = 3–4) and *SMNΔ7* mice (N = 3). (**I**) PCP-2 staining of a cerebellar lobule (upper panel) and PCs (lower panel) from an autopsy section of a human control and an SMA type I patient (scale bars: upper = 200 μm, lower = 100 μm). (**J**) PC density (number/mm) in human controls (N = 15) and SMA Type I patients (N = 7). (**K**) Correlation between PC number and *post-mortem* interval (PMI) (upper graph) and age of the patients (lower graph) for human controls (N= 15) and SMA Type I patients (N = 7). N refers to the number of patients or mice in each group, and n refers to the number of cells analyzed. Statistical analysis was performed using multiple t-tests (B–F), two-way ANOVA (G, H), Mann–Whitney test (J), and simple linear regression (K).

To study cerebellar pathology in SMA, we examined the vermal region in the *SMNΔ7* mouse model of severe SMA^36,48^. Sagittal cross-sections of the vermis were incubated with antibodies against the Purkinje cell protein 2 (PCP-2), a specific marker of PCs^49^, followed by confocal imaging and quantification of the overall cerebellar structure and the number of PCs. This analysis revealed abnormal foliation, reduced size of the molecular, granular, and white matter layers as well as the total area of lobules VI and VII in the cerebellar vermis of *SMNΔ7* mice at disease end-stage (P10) compared to healthy littermates (Fig. 1A–E). Importantly, *SMNΔ7* mice showed selective loss of PCP-2^+^ PCs in the apical regions of lobules VI and VII (Fig. 1A, F). Longitudinal analysis revealed progressive degeneration of SMA PCs in lobules VI and VII starting at P4 and preceding foliation defects and cerebellar hypoplasia at P7 (Fig. 1A, G, H). Double labeling with PCP-2 and calbindin, another marker for PCs^49^, confirmed that the observed changes were due to PC degeneration rather than selective downregulation of PCP-2 in severe P10 SMA mice (**Fig. S1A**).

To determine whether PC loss is also a feature of SMA in humans, we quantified PCs in vermal cerebellar regions of 15 control individuals and 7 SMA Type I patients (**Supplementary Table 1**). Although the identity of specific lobules could not be determined from cerebellar autopsy tissue, we found an overall 35% reduction in PC density in the cerebellum of SMA patients relative to controls (Fig. 1I, J). Importantly, the observed reduction of PC number was independent of the *postmortem* interval (PMI) and age of individuals (Fig. 1K).

To determine whether PC death is a shared feature of motor neuron diseases, we investigated the cerebellum of milder *Smn*^*2B/-*^ SMA mice as well as *SOD1-G93A* and *Ighmbp2*^*NMD−2J*^ mice as models of familial ALS and SMA with respiratory distress type 1 (SMARD1), respectively. None of these models exhibited PC loss or cerebellar alterations at disease end-stage (**Fig. S1B–G**), indicating that PC degeneration and cerebellar hypoplasia are specific features of severe SMA that are not present in other motor neuron diseases.

Together, these results identify selective and progressive death of PCs as a disease feature in a severe mouse model of SMA that is validated in *post-mortem* tissue from Type I SMA patients.

### Functional output PCs is altered in *SMNΔ7* mice

**Figure 2:**
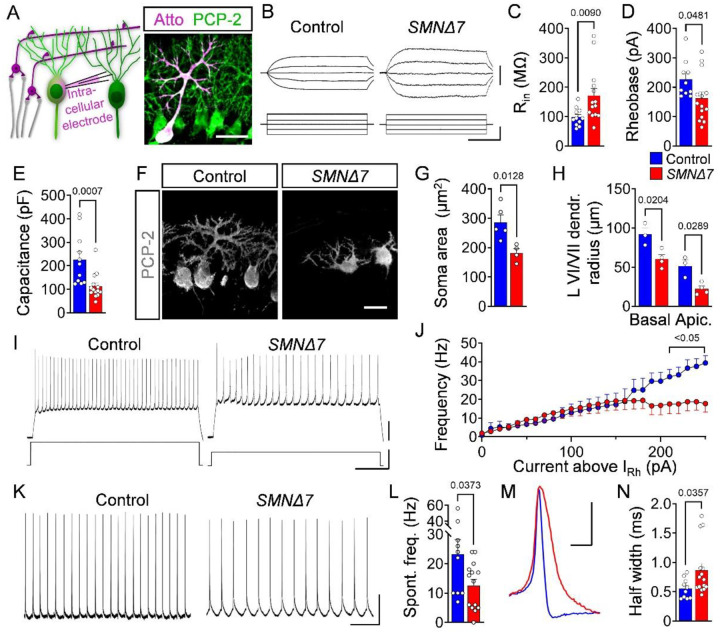
Functional output of PCs is altered in SMA mice. (**A**) Schematic of the whole-cell patch-clamp approach for measurement of PCs (left panel). Confocal image of a PC filled with Atto (magenta) during recording, co-stained with PCP-2 (green) (right panel) (scale bar = 50 μm). (**B**) Membrane responses following current injections of PCs in lobules VI/VII from P10 control and *SMNΔ7* animals (scale bars = 5 mV, 50 pA, and 100 ms). Quantification of (**C**) input resistance R_in_, (**D**) rheobase, and (**E**) capacitance for control (n = 13) and *SMNΔ7* (n = 14) PCs from at least N = 4 mice per genotype at P10. (**F**) PCP-2 staining and (**G**) quantification of the PC soma area from control (N = 5) and *SMNΔ7* (N = 4) mice (scale bar = 20 μm). (**H**) Quantification of dendritic tree radius for basal and apical PCs of lobules VI/VII from control (N = 3) and *SMNΔ7* (N = 5) mice. (**I**) Traces of induced firing rate of PCs of lobules VI/VII from a P10 control and *SMNΔ7* mouse (scale bars: 20 mV, 400 pA, and 200 ms). (**J**) Quantification of firing frequency depending on the current injection above the rheobase in control (n = 13) and *SMNΔ7* (n = 14) PCs from at least N = 4 mice per genotype at P10. (**K**) Traces of spontaneous firing of control and *SMNΔ7* PCs at P10 (scale bars = 20 mV and 200 ms). (**L**) Quantification of spontaneous firing frequency in control (n = 13) and *SMNΔ7* (n = 14) PCs from at least N = 4 mice per genotype at P10. (**M**) Example of PC action potential and (**N**) quantification of the half-width of control (n = 13) and *SMNΔ7* (n = 14) PCs from at least N = 4 mice per genotype at P10 (scale bar = 20 mV, 200 ms). Statistical analysis was performed using unpaired t-test (C–E, G, H, L, N).

To assess PC function in SMA mice, we recorded intracellularly from vulnerable PCs of lobules VI/VII at early (P5) and late (P10) stages of cerebellar pathology (Fig. 2A). At P5, we found no significant differences in input resistance (R_in_), time constant (τ) and capacitance of SMA PCs (**Fig. S2A-D**), suggesting neither changes in their passive membrane properties nor in their morphology occur at the onset of degeneration. Moreover, while these SMA PCs exhibited no alteration in rheobase, they had lower resting potential (RMP) and lower voltage firing threshold (V_thr_) (**Fig. S2E-G**). The action potentials (APs) of SMA PCs were narrower with a mildly increased amplitude compared to controls (**Fig. S2H-J**). In agreement, SMA PCs showed significantly higher evoked firing frequencies (**Fig. S2K, L**) and a tendency toward increased spontaneous firing (**Fig. S2M**), suggesting that an increased output of the cerebellar cortex may take place before substantial loss of PCs.

At a later disease stage (P10), SMA PCs were hyperexcitable as indicated by an increased R_in_ and decreased rheobase (Fig. 2B–D), while RMP, V_thr_ and AP amplitude were unaltered (**Fig. S2N-P**). A significantly decreased capacitance and unchanged τ are indicative of smaller soma size and proximal dendritic trees in vulnerable SMA PCs from lobules VI/VII (Fig. 2E **and Fig. S2Q**), which agrees with the morphological analysis (Fig. 2F–H). Thus, the observed hyperexcitability likely reflects the decreased size of PCs. In contrast, PCs located in lobule III, which are resistant to death, showed no morphological signs of impairment (**Fig. S2R**). The evoked as well as the spontaneous AP firing rate of PCs were reduced by ~50% in SMA mice relative to control littermates at P10 (Fig. 2I–L). The paradox of hyperexcitability and reduced functional output could be explained by the increased AP half-width, which was significantly wider in the repolarizing phase of SMA PCs at P10 (Fig. 2M, N). These results indicate a decreased functional output of degenerating PCs at disease end-stage in SMA mice.

Together, our findings indicate that the functional output of the cerebellar cortex is increased at an early stage of disease prior to PC loss, but it is significantly reduced at later stages concomitant with PC death in SMA mice.

### Synaptic inputs onto PCs are reduced and dysfunctional in *SMNΔ7* mice

**Figure 3:**
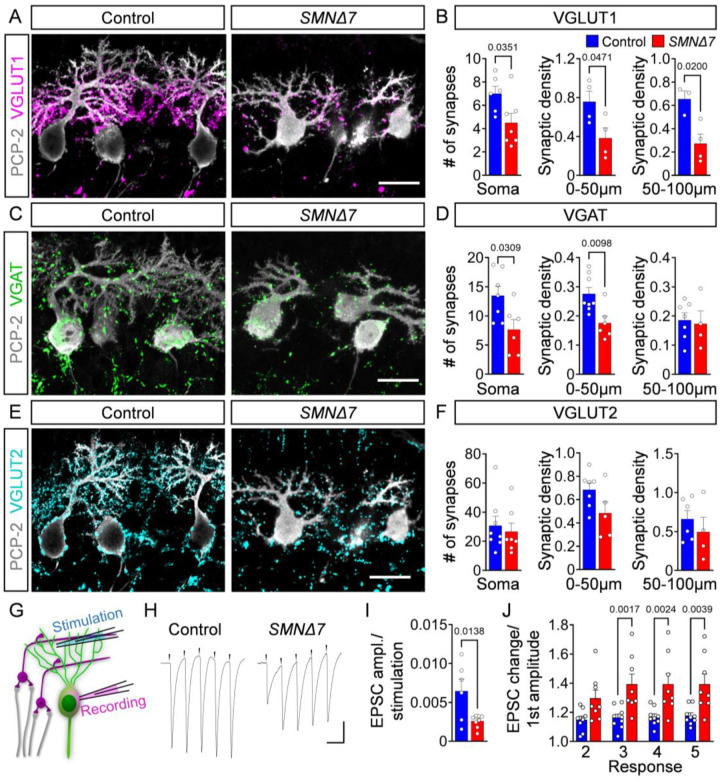
Synaptic inputs onto PCs are reduced and dysfunctional in SMA. (**A**) Immunostaining of PCP-2 (grey) and VGLUT1 (magenta) in control and *SMNΔ7* apical PCs from lobules VI/VII at P10 (scale bar = 20 μm). (**B**) Quantification of VGLUT1 synapses on apical PC soma, and proximal (0–50 μm) and distal (50–100 μm) dendrites in lobules VI/VII of control (N = 3–6) and *SMNΔ7* (N = 4–7) mice. (**C**) Immunostaining of PCP-2 (grey) and VGAT (green) in control and *SMNΔ7* apical PCs from lobules VI/VII at P10 (scale bar = 20 μm). (**D**) Quantification of VGAT synapses on apical PC soma, and proximal (0–50 μm), and distal (50–100 μm) dendrites in lobules VI/VII of control (N = 6–8) and *SMNΔ7* (N = 4–6) mice. (**E**) Immunostaining of PCP-2 (grey) and VGLUT2 (cyan) in control and *SMNΔ7* apical PCs from lobules VI/VII at P10 (scale bar = 20 μm). (**F**) Quantification of VGLUT2 synapses on apical PC soma, and proximal (0–50 μm) and distal (50–100 μm) dendrites from lobules VI/VII of control (N = 6–8) and *SMNΔ7* (N = 4–6) mice. (**G**) Schematic of the approach for EPSC measurements in PCs following parallel fiber stimulation. (**H**) Representative traces of EPSCs of P10 control and *SMNΔ7* PCs from lobules VI/VII following 200 Hz parallel fiber stimulation (scale bars = 100 pA and 5 ms). (**I**) Ratio of EPSC amplitude/stimulation in control (n = 9) and *SMNΔ7* (n = 8) PCs in lobules VI/VII and stimulation intensity for parallel fibers. (**J**) Quantification of EPSC amplitude changes of the second, third, fourth, and fifth responses normalized to the first response of P10 control (n = 9) and *SMNΔ7* (n = 8) PCs from lobules VI/VII following parallel fiber stimulation. Statistical analysis was performed using unpaired t-test (B, D, F, I) and two-way ANOVA (J).

The functional output of PCs is shaped by the activity of excitatory and inhibitory cerebellar neurons. Therefore, we investigated whether cerebellar circuit dysfunction could contribute to alter PC output in SMA mice. To do so, we used antibodies against vesicular GABA transporter (VGAT) as a marker for inhibitory synapses originating from basket, stellate, and Golgi neurons^36^ as well as neighbouring PC cells^50,51^ (**Fig. S3A**). To visualize excitatory synapses, we used antibodies against vesicular glutamate transport proteins (VGLUT1 and VGLUT2) as markers of parallel fiber terminals originating from GCs and climbing fibers from the inferior olive^52^. During development, parallel fibers transition from expressing VGLUT2 to VGLUT1 after the first postnatal week^53^. Consistent with this, we observed small parallel fiber synapses exclusively expressing VGLUT1 on the soma and proximal dendrites of PCs, whereas larger VGLUT2^+^ climbing fiber synapses were found contacting the soma and primary dendritic shafts in P10 mice (**Fig. S3B**). We investigated the apical regions of the vulnerable lobules VI/VII, which exhibited PC death, and the resistant lobule III, which has no detectable PC loss (Fig. 1A, F). VGLUT1^+^ parallel fiber synapses were reduced by ~30% on the soma and ~50% on both proximal (0–50 μm from the soma) and distal (50–100 μm) dendrites of PCs in the vulnerable lobules VI and VII of *SMNΔ7* mice at P10 (Fig. 3A, B), whereas resistant PCs in lobule III showed no reduction (**Fig. S4A, B**). Inhibitory VGAT^+^ synapses were also selectively reduced albeit to a lesser extent (~30% decrease onto the soma and proximal dendrites but unchanged onto distal dendrites) in vulnerable PCs of lobules VI/VII (Fig. 3C, D **and Fig. S4C-D**). In contrast, VGLUT2^+^ synapses from climbing fibers were spared or only mildly affected in PCs of vulnerable and resistant lobules of *SMNΔ7* mice (Fig. 3E, F **and Fig. S4E-F**). To test whether synaptic loss is limited to the severe form of SMA, we investigated synaptic density onto PCs in the milder *Smn*^*2B/-*^ mouse model and found that all three types of synapses were unaltered at disease end-stage (**Fig. S4G, H**). Together, these findings reveal a substantial loss of excitatory parallel fibers and a milder reduction of inhibitory synapses onto vulnerable PCs in severe SMA mice.

Next, we investigated the impact of synaptic dysregulation on neurotransmission by recording intracellular excitatory post-synaptic currents (EPSCs) from PCs following extracellular stimulation of parallel fibers (Fig. 3G). We normalized the EPSC amplitude of the PCs to the stimulation current injected into the parallel fibers. EPSCs from PCs of the vulnerable lobules VI/VII in *SMNΔ7* mice were reduced by ~50% (Fig. 3H, I), consistent with dysfunction and loss of VGLUT1^+^ synapses from parallel fibers (Fig. 3B). To dissect pre- and postsynaptic mechanisms for the weakening of parallel fiber synapeses, we measured the short-term plasticity. The EPSC amplitudes normalized to the first EPSC amplitude at the parallel fiber to PC synapses in lobules VI/VII increased from ~20% in control mice to ~40% in SMA mice (Fig. 3H, J). The profound increase in the degree of synaptic facilitation indicates a reduced presynaptic release probability, which is consistent with the lower EPSC amplitudes as well as the increased facilitation due to less vesicle depletion^54^. In contrast, SMA PCs in the resistant lobule III did not show differences in facilitation or EPSC amplitudes compared to control PCs (**Fig. S4I-K**). Thus, the substantial loss of excitatory parallel fiber synapses may be further aggravated by reduced presynaptic neurotransmission in vulnerable lobules of severe SMA mice.

To further evaluate the functional input of the cerebellum, we recorded the synaptic transmission between mossy fibers - originating from different nuclei of the vestibular system, cerebellar cortex, reticular formation, and spinal cord^36^ - and the GCs in the cerebellar cortex as the source of the parallel fibers. Although SMA GCs in vulnerable lobules had slightly smaller and wider AP (**Fig. S5A-D**), all other passive and active functional properties were unaltered compared to control GCs (**Fig. S5E-N**). Moreover, repetitive 100-Hz-stimulation of mossy fibers resulted in similar short time depression in control and SMA GCs (Fig. 5O–R), indicating that the mossy fiber to GC synapses, which mediate the main sensory input to the cerebellar cortex, is unaltered in SMA.

Together, these findings highlight impairment of excitatory neurotransmission and degeneration of parallel fiber boutons, leading to reduced activation of PCs and diminished cerebellar output in SMA.

### USV is impaired in *SMNΔ7* mice

**Figure 4:**
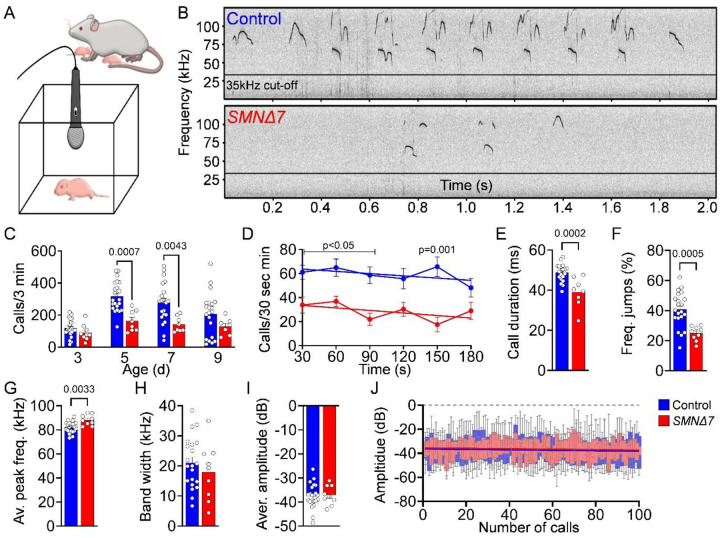
Severe SMA mouse model show decreased USV production. (**A**) Schematic of the experimental setup for recording USV from isolated mouse pups. (**B**) Representative USV spectograms from control and *SMNΔ7* mice at P5. (**C**) Quantification of the number of calls per 3 minutes in control (N = 22) and *SMNΔ7* (N = 8) mice at P3, P5, P7, and P9. Acoustic features analysis of USVs in control (N = 22) and *SMNΔ7* (N = 8) mice at P5: (**D**) call number per 30 seconds during a 3 minutes recording period (linear regression, p-values for slope equality = 0.9361), (**E**) call duration, (**F**) frequency jumps, (**G**) average peak frequency at maximum amplitude, (**H**) average bandwidth per call, (**I**) average of maximum amplitude per call and (**J**) maximum amplitude of the first 100 calls (linear regression, p-values for slope equality = 0.9921). Statistical analysis was performed using two-way ANOVA (C), multiple t-test (D) and unpaired t-test (E-I), simple line regression comparison (D, J).

Next, we sought out to identify potential behavioral consequences of cerebellar pathology in SMA mice. Interestingly, PC degeneration and hypoplasia specifically confined to vermal lobules VI and VII have previously been associated with neurodevelopmental delay in motor function, speech and cognition in both humans and mice^55–60^. To assess neurodevelopmental delay in *SMNΔ7* pups, we evaluated separation-induced ultrasonic vocalizations (USVs) (Fig. 4A), which are functionally important signals that elicit maternal care. Deficits in USVs have been reported in various rodent models of neurodevelopmental disorders, particularly those modeling human communication impairments such as verbal dyspraxia and ASD^37,61–63^. We found that the number of USV calls per 3 minutes in control mice peaked between P5 and P7 following a canonical inverted-U shape trajectory from P3 to P9 (Fig. 4B, C), which is characteristic of normal WT pups^63,64^. Conversely, SMA mice exhibited pronounced USV deficits, including an approximately 50% reduction in call number at P5 and P7 (Fig. 4B, C), displaying a stagnant trajectory reminiscent of that observed in ASD mouse models^65,66^. Further analysis of the USV acoustic properties in P5 SMA mice revealed a consistent reduced call rate over the time of recording, shorter call durations, smaller frequency jumps, and an overall higher frequency compared to control littermates (Fig. 4D–G), suggesting short, high-pitched communication with reduced vocal complexity. In contrast, the frequency range (bandwidth), average of the maximum amplitude per call and maximum amplitude during 100 calls remained unchanged in SMA mice (Fig. 4H–J), indicating a normal vocalization without fatigue despite severe motor deficits. Overall, these findings reveal neurodevelopmental deficits in the acquisition of social communication behaviors in SMA mice.

### Cerebellar pathology is independent of spinal sensory-motor circuit dysfunction in *SMNΔ7* mice

**Figure 5:**
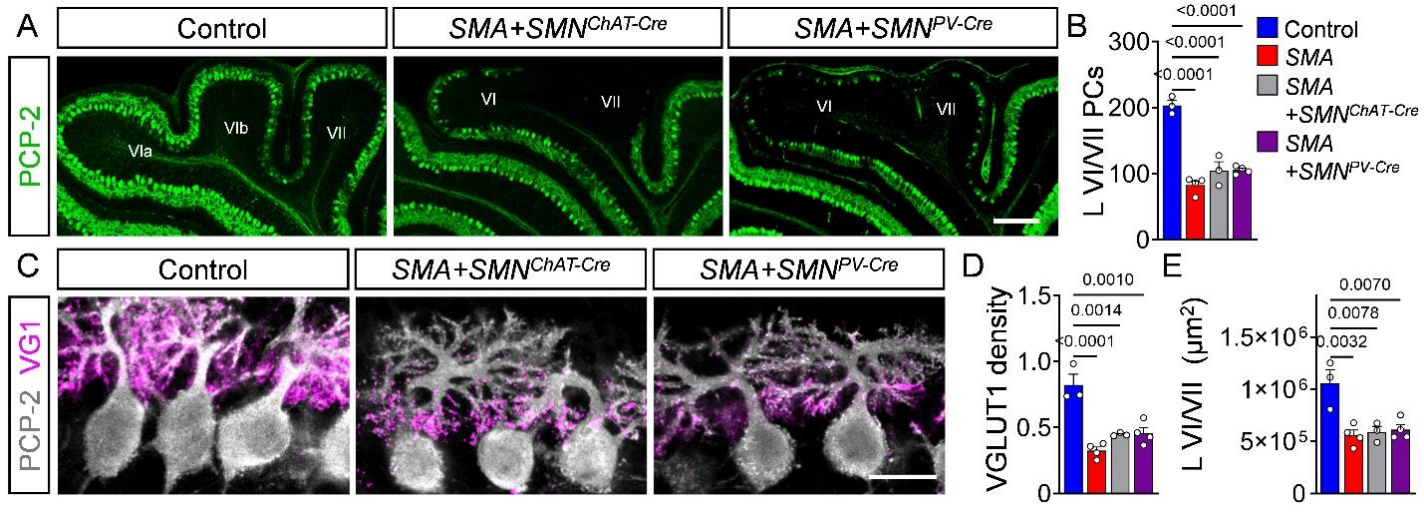
Cerebellar pathology is independent of spinal sensory-motor circuit dysfunction. (**A**) Immunostaining of PCP-2 in PCs from lobules VI/VII of control, *SMA+SMN*^*ChAT-Cre*^ and *SMA+SMN*^*PV-Cre*^ mice (scale bar: upper panel = 200 μm, lower panel = 50 μm). (**B**) Quantification of PC number in lobules VI/VII from control (N = 3), *SMA* (N = 4), *SMA+SMN*^*ChAT-Cre*^ (N = 3) and *SMA*+SMN^PV-Cre^ (N = 4) mice. (**C**) Immunostaining of PCP-2 (grey) and VGLUT1 (magenta) in PCs from control, *SMA*+*SMN*^*ChAT-Cre*^ and *SMA*+*SMN*^*PV-Cre*^ mice (scale bar = 20 μm). Quantification of (**D**) VGLUT1 dendritic density and (**E**) area of apical lobules VI/VII from control (N=3), *SMA* (N = 4), *SMA+SMN*^*ChAT-Cre*^ (N=3) and *SMA+SMN*^*PV-Cre*^ (N = 4) mice. Statistical analysis was performed using one-way ANOVA.

The cerebellum plays a crucial role in processing proprioceptive information and modulating motor output, which are both strongly affected in SMA mouse models and patients^5,21,22,24,34,35,67^. This raises the question whether cerebellar degeneration is a consequence of spinal sensory-motor circuit pathology in SMA. To address this, we first aimed to rescue SMA specifically in motor neurons by exploiting a previously established mouse model for conditional rescue of SMA (*SMA*^*Res*^) harboring two homozygous human transgenes (*SMN2* and *SMN*Δ*7*), one *Smn* null allele (Smn^KO^), and one Cre-inducible *Smn* allele (*Smn*^*Res*^) with a switch cassette flanked by loxP sites in opposing orientation^68^. In the absence of Cre recombinase, the phenotype of the *SMA*^*Res*^ model is similar to that of *SMN*Δ*7* mice because the mouse/human *Smn*^*Res*^ hybrid allele produces predominantly a truncated, non-functional SMN protein. However, Cre-mediated genomic recombination of the switch cassette leads to the expression of full-length, functional SMN^21,26,27,68^. We and others have previously demonstrated that *SMA*^*Res*^ mice harboring Choline acetyltransferase (ChAT)-Cre exhibit rescue of pathology in motor neurons (*SMA+SMN*^*ChAT-Cre*^)^21,26,27^. The rescue of the pathology in motor neurons in of *SMA+SMN*^*ChAT-Cre*^ mice did not rescue the cerebellar pathology including the lobule area, PC number, and density of VGLUT1^+^ parallel fiber terminals in vulnerable lobules VI/VII (Fig. 5), demonstrating that the cerebellar alterations are independent of motor neuron pathology.

To address whether sensory defects contribute to the cerebellar pathology in SMA, we restored SMN in proprioceptive neurons using a parvalbumin (PV)-Cre driver in *SMA+SMN*^*PV-Cre*^ mice, which exhibit rescued proprioceptive function^21^. We leveraged the fact that developing PCs in lobules VI/VII do not express parvalbumin until the second postnatal week, in contrast to the early expression of both PCP-2 and calbindin (**Fig. S6**) confirming previously reports in wild-type mice^69^. Consequently, SMN cannot be restored in these vulnerable PCs prior to their degeneration in *SMA+SMN*^*PV-Cre*^ mice. Notably, the rescue of the proprioceptive pathology of *SMA+SMN*^*PV-Cre*^ mice did not rescue any aspect of the cerebellar pathology (Fig. 5), effectively uncoupling the observed cerebellar alterations from proprioceptive dysfunction.

Taken together, the persistence of cerebellar pathology despite SMN rescue in motor or proprioceptive neurons indicates that PC and parallel fiber bouton degeneration occurs independently of spinal sensory-motor circuit defects in SMA mice.

### Activation of the p53 pathway drives PC degeneration in *SMNΔ7* mice

**Figure 6:**
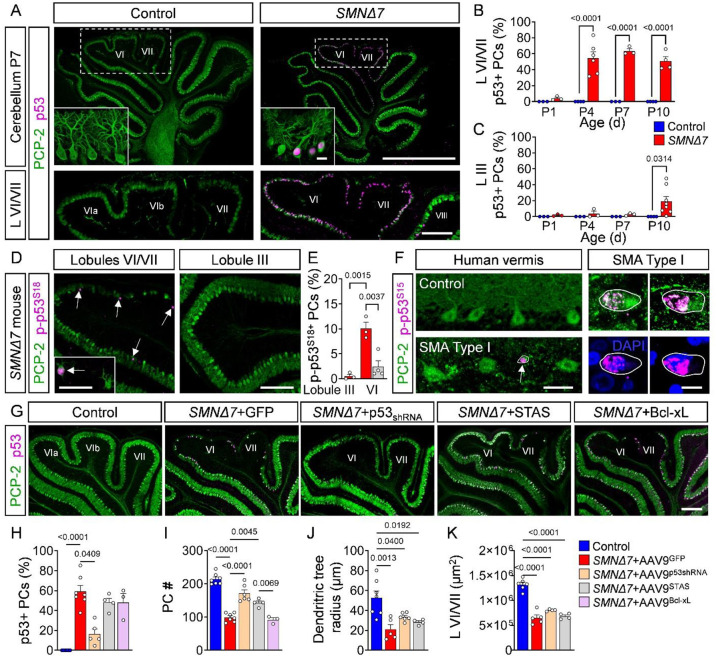
Converging p53 upregulation and phosphorylation causes PC death. (**A**) Immunostaining of PCP-2 (green) and p53 (magenta) in cerebellar sections and lobules VI/VII of control and *SMNΔ7* mice at P7 (scale bars: upper panel = 1 mm, inset = 20 μm; lower panel = 200 μm). Quantification of p53^+^ PCs in % over time in (**B**) lobules VI/VII and (**C**) lobule III from control (N = 4) and *SMNΔ7* (N = 4) mice. (**D**) Immunostaining of PCP-2 (green) and p-p53^S18^ (magenta) in lobules III and VI/VII from *SMNΔ7* mice at P10 (scale bar = 200 μm, inset = 50 μm). (**E**) Quantification of p-p53^S18+^ PCs in % of lobules III and VI/VII from *SMNΔ7* mice (N = 3) (red bars) and *SMNΔ7*+AAV9-STAS mice (N = 4) (grey bar) at P10. (**F**) Immunostaining of p-p53^S18^ (magenta) and PCP-2 (green) or DAPI (blue) in the human vermis of a control and SMA Type I autopsy tissue (circle and arrow indicate p-p53^S15+^ degenerating PCs; scale bar: left = 50 μm, right = 10 μm). (**G**) Immunostaining of PCP-2 (green) and p53 (magenta) in lobules VI/VII of control, *SMNΔ7*+AAV9-GFP, *SMNΔ7*+AAV-p53_shRNA_, *SMNΔ7*+AAV9-STAS and *SMNΔ7*+AAV9-Bcl-xL mice at P10 (scale bar = 200 μm). Quantification of (**H**) p53^+^ PCs in %, (**I**) total number of PCs, (**J**) dendritic tree radius of PCs and (**K**) area of lobule VI/VII of control (N = 6–7), *SMNΔ7*+AAV9-GFP (N = 6), *SMNΔ7*+AAV9-p53_shRNA_ (N = 5–7), *SMNΔ7*+AAV9-STAS (N = 4–6), and *SMNΔ7*+AAV9-Bcl-xL (N = 3) mice. Statistical analysis was performed using two-way ANOVA (B, C) and one-way ANOVA (H-K).

To investigate potential intrinsic drivers of this cerebellar vulnerability, we examined whether p53-mediated mechanisms—previously implicated in motor neuron degeneration in SMA mouse models^18–20,24,70^—also contribute to PC loss. First, we first performed a longitudinal analysis of p53 expression in the cerebellum of SMNΔ7 mice using immunohistochemistry and confocal microscopy. While control littermates did not show any p53 expression in the cerebellum as expected, *SMNΔ7* mice exhibited strong, time-dependent nuclear accumulation of p53 in PCs (Fig. 6A–C). Importantly, the proportion of p53^+^ SMA PCs in lobules VI/VII increased from nearly none at P1 to ~50% at P4, coinciding with the onset of PC degeneration (Fig. 1H and Fig. 6B). In contrast, resistant PCs of lobule III (and other lobules) showed p53 expression only at disease end-stage (~20%) (Fig. 6C).

In addition to p53 upregulation^18,20^, phosphorylation of the amino-terminal transactivation domain of p53 including serine 18 (S18) (corresponding to serine 15 in human p53) is a necessary event to induce death of motor neurons in SMA mice^18,19^. Therefore, we investigated the expression of p53 phosphorylated at S18 (p-p53^S18^) by immunohistochemistry with phosphor-specific antibodies in the cerebellum of *SMNΔ7* mice. Notably, the expression of p-p53^S18+^ was confined to vulnerable PCs in lobules VI/VII and was absent in resistant PCs in lobule III at P10 (Fig. 6D, E). Moreover, the majority (~80%) of PCs from the intermediate *Smn*^*2B/-*^ mouse model of SMA was p53^+^ but did not express p-p53^S18^ at disease end-stage (**Fig. S7A-D**), in agreement with the lack of PC death in this model (**Fig. S1F, G**). These results identify nuclear accumulation of p-p53^S18^ as a marker for degenerating PCs in *SMNΔ7* mice, akin to the situation in SMA motor neurons^18^.

To evaluated whether p53 activation contributes to PC loss in human SMA pathology, we performed immunohistochemistry experiments with p-p53^S15^ antibodies and well-preserved cerebellar sections from a control individual and a Type I SMA patient. While we found no p-p53^S15^ immunoreactivity in control tissue, nuclear p-p53^S15^ accumulation was detected in small degenerating PCs of the SMA cerebellum (Fig. 6F **and Fig. S7E**). Consistent with our findings in *SMNΔ7* mice, these results point to the induction of p-p53^S15^ as a conserved event and a candidate marker of PC degeneration in SMA patients.

To determine whether p53 activation drives the death of SMA PCs, newborn *SMNΔ7* mice were injected intracerebroventricularly (ICV) with a self-complementary adeno-associated virus serotype 9 (AAV9) expressing GFP and a short hairpin RNA (shRNA) against mouse p53, which was previously validated to knockdown p53 *in vivo*^18^, or GFP alone as a control. AAV9-p53_shRNA_ exclusively transduced PCs in the cerebellum with ~70% efficacy measured by GFP expression (**Fig. S7F, H**). Importantly, injection of AAV9-p53_shRNA_ but not AAV9-GFP strongly reduced nuclear p53 staining (Fig. 6G, H) and increased the number of PCs in lobules VI/VII of *SMNΔ7* mice (Fig. 6G, I), demonstrating that p53 activation drives selective PC death in SMA mice.

Previous work showed that SMN-dependent splicing dysregulation of *Stasimon* – an essential ER resident transmembrane protein^71,72^ – contributes to p53^S18^ phosphorylation and motor neuron death in SMA models^19,32^. To determine whether *Stasimon* dysfunction is linked to the death of SMA PCs, we ICV injected *SMNΔ7* mice at P0 with a previously described AAV9 vector driving *Stasimon* expression (AAV9-STAS)^19^. Notably, *Stasimon* gene delivery reduced the percentage of PCs expressing p-p53^S18^ (Fig. 6E), did not alter the nuclear accumulation of p53 (Fig. 6G, H), and significantly increased the number of PCs compared to AAV9-GFP treatment in SMA mice at P10 (Fig. 6G, I). In contrast, gene delivery of either AAV9-p53_shRNA_, or AAV9-STAS did neither improve PC shape nor lobule size in *SMNΔ7* mice (Fig. 6J, K). These results highlight the contribution of *Stasimon* dysfunction to the amino-terminal phosphorylation of p53 and selective death of PCs in SMA mice.

A well-established pathway by which p53 activation executes cell death is through apoptosis^73^. Additionally, multiple studies have demonstrated that the overexpression of Bcl-xL protects neurons from p53-induced apoptosis by preventing the release of mitochondrial cytochrome c and caspase activation^74^. To determine whether p53 drives PC death in SMA mice through apoptosis, we injected *SMNΔ7* mice with a previously validated AAV9 vector expressing Bcl-xL fused to mCherry (AAV9-Bcl-xL)^75^. Interestingly, robust expression of mCherry-Bcl-xL in ~90% of SMA PCs did neither alter p53 expression nor rescue these neurons from degeneration (Fig. 6G–I **and Fig. S7G, H**), suggesting that p53 may be acting through a non-apoptotic downstream pathway.

Collectively, these findings indicate that converging mechanisms of p53 activation, including its upregulation and amino-terminal phosphorylation, drive PC death via non-apoptotic mechanisms in *SMNΔ7* mice and possibly severe SMA patients.

### SMN restoration in PCs prevents their death and improves neurodevelopmental deficits in *SMNΔ7* mice

To determine whether intrinsic effects of SMN deficiency in PC contribute to cerebellar pathology and the SMA phenotype, we established AAV9 vectors for selective expression in PCs of either GFP (AAV9-L7-6-GFP) or human SMN (AAV9-L7-6-SMN) driven by a previously characterized, PC-specific minimal promoter (L7–6)^76^. First, we analyzed GFP expression to determine the onset and cell-specific expression of this viral approach. SMA mice injected ICV at P0 with AAV9-L7-6-GFP exhibited highly selective expression of GFP in ~90% of PCs as early as 24 hours after injection, while no GFP was detectable in the cerebrum, dorsal root ganglions (DRGs), spinal cord, and muscles up to 10 days after injection (**Fig. S8A-E**). To assess the efficacy and selectivity of SMN restoration by AAV9-L7-6-SMN, we utilized immunohistochemistry to visualize SMN-containing nuclear structures known as gemini of coiled bodies (gems)^77^ as a readout for SMN expression. Gems were observed in ~90% of PCs from SMA mice injected with AAV9-L7-6-SMN, whereas less than 1% of PCs contained gems in SMA mice treated with AAV9-L7-6-GFP (**Fig. S8F, G**). In contrast, AAV9-L7-6-SMN did not elevate SMN expression in vulnerable lumbar L1 motor neurons of SMA mice. (**Fig. S8H, I**). Together, these findings highlight selective and efficient restoration of SMN in SMA PCs following transduction with our viral-mediated approach.

**Figure 7:**
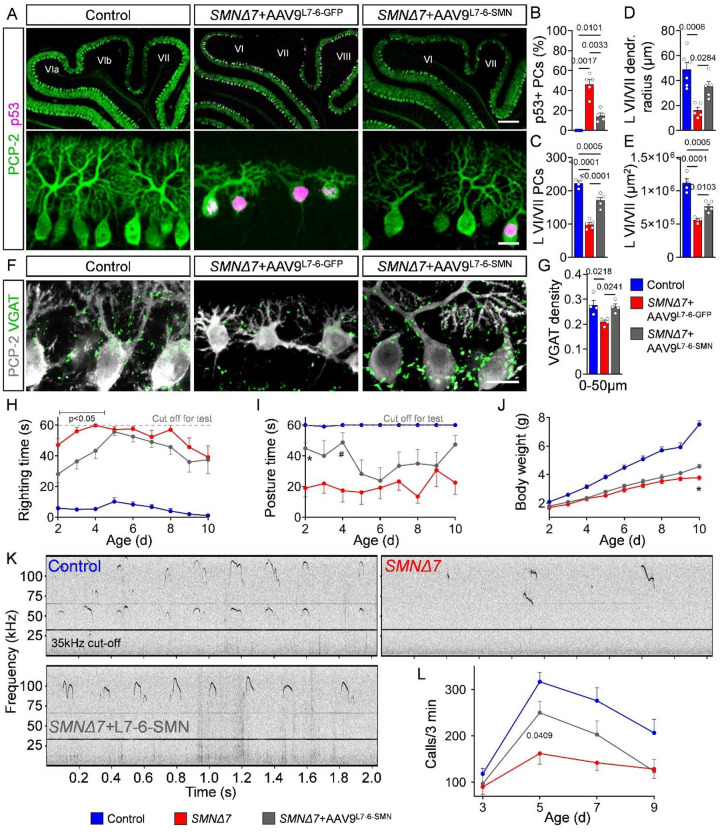
Cell-autonomous PC death contributes to motor and communication defects. (**A**) Immunostaining of PCP-2 and p53 in PCs from lobules VI/VII of control, *SMNΔ7*+AAV9-L7-6-GFP and *SMNΔ7*+AAV9-L7-6-SMN mice at P10 (scale bars: upper panel = 200 μm, lower panel = 20 μm). Quantification of (**B**) p53^+^ PCs, (**C**) total number of PCs, (**D**) dendritic radius of PCs and (**E**) area of lobules VI/VII in control (N = 6), *SMNΔ7*+AAV9-L7-6-GFP (N = 5) and *SMNΔ7*+AAV9-L7-6-SMN (N = 5) mice. (**F**) Immunostaining of PCP-2 (grey) and VGAT (green) in lobules VI/VII from control, *SMNΔ7*+AAV9-L7-6-GFP and *SMNΔ7*+AAV9-L7-6-SMN mice at P10 (scale bar = 20 μm). (**G**) Quantification of dendritic synaptic density (0–50 μm from the soma) of VGAT^+^ synapses onto lobules VI/VII PCs of control (N = 6), *SMNΔ7*+AAV9-L7-6-GFP (N = 5) and *SMNΔ7*+AAV9-L7-6-SMN (N=5) mice at P10. (**H**) Righting time, (**I**) posture time and (**J**) body weight of control (RT: N=18, PT: N=13, BW: N=18), *SMNΔ7*+AAV9-L7-6-GFP (RT: N = 11, PT: N = 7, BW: N = 11) and *SMNΔ7*+AAV9-L7-6-SMN (RT: N = 13, PT: N = 7, BW: N = 13) mice. (**K**) Representative audio tracks from P5 control, *SMNΔ7* and *SMNΔ7*+AAV9-L7-6-SMN mice. **(L)** Quantification of the number of calls per 3 minutes in control (N = 22), *SMNΔ7* (N = 8) and *SMNΔ7*+AAV9-L7-6-SMN (N = 8) mice at P3, P5, P7, and P9. Note that the data for control and SMA mice are the same as presented in Fig. 4, the AAV9-L7-6-SMN–treated mutants were littermates of the other two groups, and all experiments were conducted at the same time. Statistical analysis was performed using one-way ANOVA (B, C, D, E, G) and two-way ANOVA (H-J, L), p-values for I: * = 0.0072, # = 0.0202 and J: * = 0.0108 comparing *SMNΔ7*+AAV9-L7-6-GFP with *SMNΔ7*+AAV9-L7-6-SMN.

Next, we investigated the impact of restoring SMN selectively in PCs on cerebellar circuit pathology. Treatment with AAV9-L7-6-SMN, but not with AAV9-L7-6-GFP, led to a significant reduction in the percentage of p53^+^ PCs and increased PC numbers in SMA mice (Fig. 7A–C), strengthening the link between SMN-dependent p53 activation and cell-autonomous PC death. SMN restoration in SMA PCs also enhanced dendritic arborization and mildly mitigated cerebellar hypoplasia in lobules VI/VII (Fig. 7D, E). Furthermore, AAV9-L7-6-SMN improved the number of inhibitory VGAT^+^ synapses likely originating from neighboring rescued PCs (Fig. 7F, G **and Fig. S3A**). In contrast, the loss of excitatory VGLUT1^+^ synapses from parallel fibers onto PCs was not reversed by AAV9-L7-6-SMN (**Fig. S9A-C**), highlighting the non-cell-autonomous origin of this defect. These improvements were cerebellum-specific as AAV9-L7-6-SMN treatment did not ameliorate spinal cord pathology, including p53-mediated motor neuron death, NMJ denervation, and loss of proprioceptive synapses (**Fig. S9D-K**). Thus, SMN deficiency triggers PC death through mechanisms that involve cell-autonomous p53 activation and are independent from degeneration of parallel fiber boutons.

Lastly, we asked whether PC degeneration contributes to neurodevelopmental motor and vocalization deficits in SMA mice. We found that selective SMN restoration in PCs moderately improved gross motor performance as assessed by righting reflex and body posture assays until P4, while it had essentially no effects on weight gain and survival in SMA mice (Fig. 7H–J **and Fig. S9L**), indicating a cerebellar involvement in the early postnatal motor phenotype of SMA. We also investigated the effects of AAV9-L7-6-SMN treatment on the vocalization deficits of SMA mice by performing USV experiments. We found that selective restoration of SMN in SMA PCs increased the number of USV calls per 3 minutes at the P5 and P7 time points relative to untreated *SMNΔ7* mice (Fig. 7K–L), re-establishing the inverted U-shape profile that is characteristic of normal early postnatal development but strongly blunted in SMA mice. In contrast, other acoustic features such as call duration, frequency jumps, and peak frequency were not improved by AAV9-L7-6-SMN gene delivery in SMA mice (**Fig. S9M-O**). These findings demonstrate that cell-autonomous degeneration of PCs induced by SMN deficiency contributes to neurodevelopmental deficits in SMA mice.

## Discussion

Motor impairments have historically taken center stage in SMA research and have previously been linked to the dysfunction of spinal motor circuits. Following the advent of disease modifying therapies, which extend survival and significantly, yet partially, improve motor function in SMA patients, there has been an increasing recognition of the clinical relevance of cognitive and social deficits as well as the involvement of neuronal circuits in the brain as potential disease contributors. In this context, our study highlights a novel role for cerebellar pathology in motor and communication deficits in SMA. The cerebellum plays a critical role in motor coordination, cognition, and social behavior. By integrating multiple lines of evidence for PC degeneration, circuit-level cerebellar disruption, and new neurodevelopmental phenotypes from a severe mouse model of the disease with analysis of *post-mortem* tissue from Type I patients, we highlight the cerebellum as a clinically relevant, previously underappreciated site of SMA pathology.

Our study identifies PC degeneration as a signature of SMA that is conserved across mice and humans and establishes PCs (~35% loss) as the second most vulnerable neuronal population after motor neurons (~50% loss) in SMA patients. The identification of selective PC reduction and hypoplasia of vermal lobules VI and VII in end-stage severe SMA mice complements previous reports of PC pathology in the hemispheres of severe SMA mice^42,43^ and in SMA patients^45–47,78,79^. Our longitudinal analysis also reveals that SMA PCs are initially formed and then progressively degenerate. Importantly, through viral-mediated selective restoration of SMN in PCs, which leads to improved survival and dendritic arborizations, we highlight the cell-autonomous requirement of SMN for early development, maturation, and survival of PCs.

We also addressed the intrinsic, SMN-dependent death mechanisms of vulnerable SMA PCs by showing that they display early onset, progressive nuclear accumulation of amino-terminally phosphorylated p53 (p-p53^S18^). As is the case for PC loss, this is a shared signature of degenerating PCs in both Type I SMA patients and severe SMA mice. Furthermore, we demonstrate that AAV9-mediated knockdown of p53 or overexpression of *Stasimon* prevent PC degeneration in SMA mice by suppressing upregulation and amino-terminal phosphorylation of p53, respectively. Together, these findings indicate that the SMN-dependent, RNA-mediated mechanisms driving cell-autonomous death of SMA PCs most likely parallel those at play in SMA motor neurons^18–20,23,24^. Furthermore, while p53 is a well-known mediator of apoptosis during PC development and degeneration^80,81^, we show that overexpression of the anti-apoptotic protein Bcl-xL does not prevent the loss of SMA PCs. Consistent with previous observations in SMA motor neurons^18^, these findings support the conclusion that p53-mediated neurodegeneration occurs through non-apoptotic mechanisms in SMA mice. The identification of such neurodegenerative mechanisms remains an important question to be addressed in future studies.

Beyond neuronal death of PCs, we identify cerebellar circuit dysfunction as a novel disease feature characterized by the degeneration of excitatory parallel fiber boutons and the selective loss of inhibitory synapses onto vulnerable SMA PCs in lobules VI and VII. SMN restoration in SMA PCs indicates that these inhibitory inputs originate from neighboring PCs, consistent with established local PC-to-PC inhibitory circuits^50,51^. In contrast, selective SMN restoration in SMA PCs does not rescue excitatory parallel fiber synapses from GCs, which are not targeted by this gene delivery approach. Together with direct experimental evidence uncoupling spinal cord and cerebellar pathology, we conclude that local disruption of excitatory signaling between parallel fibers and PCs is the main driver of circuit dysfunction in the SMA cerebellum. It is also likely that the observed broadening of the action potentials and reduced firing rate in SMA PCs reflects the selective loss of their afferent excitatory synapses through mechanisms analogous to those previously described for SMA motor neurons^21,22,25,34,35^. Thus, neuronal firing deficits triggered by dysfunction of local excitatory circuits emerges as a common pathogenic mechanism affecting the functional output of both spinal cord and cerebellum in SMA.

To date, a direct role for cerebellar dysfunction to the clinical phenotypes of motor neuron diseases has not been established. Here, we provide initial evidence linking cerebellar pathology to neurodevelopmental motor and vocalization deficits in neonatal SMA mice. We show that selective restoration of SMN in PCs improves righting reflexes and posture in SMA mice at early postnatal developmental stages preceding the onset of motor neuron and proprioceptive synapse degeneration^22,24^, which may obscure cerebellar-driven contributions at later times. Moreover, since disrupting parallel or climbing fiber input to PCs impairs righting reflexes in neonates^82–86^ and our viral mediated SMN restoration approach did not improve synaptic connections in the parallel fiber–PC circuit, our results may have underestimated the cerebellar contribution to motor dysfunction in SMA. Consistent with a pathogenic role of cerebellar dysfunction in SMA, we also observed transient PC hyperactivity in lobules VI and VII—a phenomenon previously associated with motor deficits in ALS^87^ and more broadly linked to impaired motor behavior and tremor^88,89^. Additionally, PC loss and hypoplasia of vermal lobules VI and VII have been linked to hypotonia, ataxia, impaired motor coordination and balance in both mice and humans^60,90–93^. Together, these findings support the contribution of cerebellar dysfunction to early motor deficits in SMA.

A key advance of our study is the discovery of communication deficits and the direct contribution of cerebellar dysfunction to this novel neurodevelopmental phenotype in SMA mice, which implicate a broader range of neural circuits beyond those controlling spinal motor neuron networks in the disease manifestations. We report that SMA mice display profound vocalization deficits as demonstrated by alterations in USV patterns during early postnatal development. USVs are well established readouts for social communicative behavior and not simply by-products of motor activity in neonatal mouse models^94,95^. In agreement, the stagnant USV trajectory in SMA mice parallels that of ASD models, which show similar communication deficits without motor impairments^65,66^. Accordingly, the absence of changes in maximum sound volume or bandwidth, along with the relative sparing of head and ventral neck muscles involved in swallowing, speech, and laryngeal movement in SMA mice^96–98^ support the conclusion that the observed USV impairment is independent from the neuromuscular phenotype. In ASD mouse models, compelling evidence implicates the cerebellum—particularly lobules VI and VII—as major contributors to USV deficits.^37,61–63,93^. In agreement with this, we demonstrate that selective restoration of SMN in PCs leads to a significant yet partial correction of the USV phenotype in SMA mice. However, while the number and the inverted-U shape developmental profile of USV calls are specifically improved by PC-specific SMN rescue, the observation that several other acoustic features remain defective implicates additional neuronal circuit deficits. Considering the well-established influence of excitatory synapses from parallel or climbing fibers on USVs in early postnatal pups^82–84^, one possibility is that loss of these synaptic inputs, which is not corrected by selective SMN rescue in PCs, contributes to the incomplete rescue of vocalization deficits in SMA mice. In addition, recent studies have shown that the anterior cingulate cortex and bulbar neurons modulate social vocalizations in mice^99^, pointing to a potentially broader involvement of neuronal networks in these neurodevelopmental deficits of SMA mice for future investigation.

Cognitive disability, severe delay of speech, poor social interaction, and diagnosis of ASD are emerging clinical features in Type I SMA patients treated with disease modifying therapies^14–17,100^. Interestingly, most of these SMA patients received the SMN-inducing antisense oligonucleotide nusinersen^15,17^, which fails to restore SMN levels in the cerebellum^101^. Furthermore, motor and social communicative deficits have been associated with focal pathology in cerebellar lobules VI and VII in aging, Alzheimer’s and Parkinson’s diseases as well as in ASD patients and mouse models^37,56–61,65,66,93,102^. Building on previous evidence and our new findings, we propose that the cerebellar pathology and communication defects identified in a severe mouse model may be linked to the cognitive and speech impairments observed specifically in Type I SMA patients.

In summary, this study uncovers a cerebellar contribution to neurodevelopmental motor and communication deficits in SMA. By delineating intrinsic and circuit-level mechanisms underlying PC degeneration and cerebellar dysfunction, our findings expand the framework of SMA pathophysiology beyond the spinal cord and implicate a new brain structure. In the future, clinical evaluations and disease modifying therapies should include the cerebellum in order to fully address the multifaceted nature of SMA and its neurodevelopmental manifestations.

## Material and Methods

### Study design

Sample size and rules for stopping data collection were determined by previous experience and preliminary data. All data was included if the experiment was technically sound (perfusion, tissue preparation, staining, recordings, imaging etc.). The endpoints for animals were selected by previous experiments and literature references. Each experiment was replicated at least three times in different animal/autopsy tissue with the exception of p-p53 staining in human tissue due to its limited availability. The research objects were to investigate cerebellar pathology in mouse models for motor neuron diseases and autopsy tissue from SMA Type I patients. The mice were randomized to treatment group, and the investigators who assessed the behavioral, histological, USV and electrophysiological outcomes were blinded to the treatment groups.

### Animal procedures

Breeding and experiments were performed in the animal facilities of Leipzig University, Leipzig, Saxony, Germany and of Columbia University, New York, NY, USA. Animal procedures were performed according to European (Council Directive 86/609/EEC) and German (Tierschutzgesetz) guidelines for the welfare of experimental animals and the regional directorate (Landesdirektion) of Leipzig for Leipzig University, and according to National Institutes of Health Guidelines on the Care and Use of Animals and approved by the Columbia Animal Care and Use Committee (IACUC) for Columbia University.

Mice were housed in a 12h/12h light/dark cycle with access to food and water *ad libitum*. The following mouse lines were used: *SMNΔ7* FVB (JAX stock #005025)^48^, *Smn*^*2B/*-^ C57BL/6 (Dr. Kothary)^103^, *SOD1-WT* B6SJL (JAX stock #002297), *SOD1-G93A* B6SJL (JAX stock #002726)^104^ and *Ighmbp2*^*NMD−2J*^ C57BL/6 (JAX stock #002521)^105^. *PV*^*Cre*^ C57Bl/6 (JAX stock #008069) and *ChAT*^*Cre*^ C57Bl/6 (JAX stock #006410) lines were crossed with *SMNΔ7* mice to generate *Pv*^*Cre tg/-*^;*Smn*^+/−^;*SMN2*^*tg/tg*^;*SMN*Δ*7*^*tg/tg*^ and Chat^Cre tg/−^;Smn^+/−^; SMN2^tg/tg^;SMNΔ7^tg/tg^ mice. These strains were bred with mice expressing a Cre-inducible Smn allele (S*MA*^*Res*^; JAX stock #007951) to generate *SMA*^*Res*^+*SMN*^*ChAT-Cre*^, *SMA*^*Res*^+*SMN*^*PV-Cre*^ or *SMA*^*Res*^ as previously described^21^.

The following primers were used for genotyping: *SMNΔ7*: forward sequence (5’ to 3’) = GATGATTCTGACATTTGGGATG, reverse sequences (5’ to 3’) = TGGCTTATCTGGAGTTTCACAA and GAGTAACAACCCGTCGGATTC (wild-type band = 325 base pairs (bp), mSmn KO = 411 bp). *Smn*^*2B/*-^: forward sequence (5’ to 3’) = TTTGGCAGACTTTAGCAGGGC, reverse sequence (5’ to 3’) = AACTCCGGGTCCTCCTTCCT (wild-type band = 500 bp, mutant = 700 bp). *SOD1-WT* and *SOD1-G93A*: forward sequence (5’ to 3’) = CATCAGCCCTAATCCATCTGA, reverse sequence (5’ to 3’) = CGCGACTAACAATCAAAGTGA (transgene = 236bp), *Ighmbp2*^*NMD−2J*^: forward sequence (5’ to 3’) = CTGGACAGAGAGAATACCTACAGACTGCTG, reverse sequence (5’ to 3’) = GTACTGCTCTGGCAGAAGACCCATGATTGC (wild-type = 500 bp, mutant = 300 bp). For genotyping of the S*MA*^*Res*^+*SMN*^*ChAT-Cre*^, *SMA*^*Res*^+*SMN*^*PV-Cre*^ mice, please see^21^.

For AAV9 gene delivery, P0 mice were anesthetized by isoflurane inhalation and injected in the right lateral ventricle of the brain with ~1 × 10^11^ genome copies of AAV9 vectors in a PBS solution containing a vital dye (Fast Green; Sigma-Aldrich).

For phenotypic analysis, *SMNΔ7* mice treated with viral interventions were monitored daily for righting time, body weight, and posture time as previously reported^34^. Each measurement was taken three times and averaged. To comply with animal welfare guidelines, mice that experienced a 25% weight loss in a single day and were unable to right themselves were euthanized. Righting time was defined as the time required for a pup to roll onto all four limbs after being placed on its back, maintaining this position for at least 3 seconds. Posture time measured how long a pup could sustain its balance while standing on all four limbs. The tests had a maximum duration of 60 seconds as indicated in the figure. Equal numbers of male and female mice were included, and data were combined, as no sex-specific differences were observed or have been previously reported in SMA.

### AAV9 vectors

The self-complementary AAV9-GFP, AAV9-STAS and AAV9-p53_shRNA_ vectors were previously described^18,19^. For virus production, AAV-vectors were generated using a three plasmid standard protocol as previously reported^106,107^ with pDGdeltaVP providing helper sequences, and a distinct rep-cap-helper plasmid with AAV9 wild-type capsid genes. Briefly, HEK293T cells were seeded in 15cm cell culture dishes and grown in DMEM+1%Pen/Strep+10%FBS till 70% confluence, and then transfected with 2μgDNA/1000bp using the PEI transfection reagent. Cells were harvested 3 days later, suspended in lysis buffer (50mM Tris-Cl, 150 mM NaCl, 5 mM MgClm with 2x Roche protease and phosphatase inhibitors cocktails added) and lysed by repeated freeze-thaw cycles; debris were cleared by centrifugation at 3000 × g for 10 min. The AAV fraction was purified from the raw cell lysate by layering on an Optiprep discontinuous gradient 60/40/20/15% in PBS+Mg/K and centrifuging at 350.000 × g 10°C for 90 min. The AAV fraction concentrated in the 40% Optiprep band was subject to de-salting in Zeba columns (Zeba Spin Desalting Columns, 10ml, 40.000 MWCO, Thermo 87772) further concentrated using centrifugal concentrators (Vivaspin 6, 100.000 MWCO Filter, GE Healthcare 28932319) at 4000 × g 4°C for 10 min, repeated till the full volume was concentrated. Viral titer was determined by qPCR with primers directed against ITR, as previously described^106^.

The plasmid for the AAV9-Bcl-xL was a kind gift from Robert Nickells. The mCherry-BclxL sequence under the control of the ubiquitous PGK promoter has been described previously^75^. The purified AAV9-Bcl-xL virus was produced by Vector Builder.

The plasmid for the AAV9-L7-6-GFP are available from AddGene (Plasmid #126462)^76^. The GFP was replaced by DNA fragments corresponding to the open reading frame of human SMN and packed into an AAV9 by Vector Builder.

### Immunostaining of murine and human tissue

For immunostaining of human cerebellar vermis, following parental- or patient-informed consent in strict observance of the legal and institutional ethical regulations, human tissues were collected at autopsy procedure at the Johns Hopkins University, Baltimore, MD, USA or was obtained from the NIH Neurobiobank at the University of Maryland, Baltimore, MD, USA (**Supplementary Table 1**). Human cerebellum tissue was cryoprotected with a 15% sucrose solution for at least 2 h until the tissue sank to the bottom of the tube. Afterwards, the tissue was transferred to a 30% sucrose solution and stored at 4°C overnight. The next day, the cerebellum tissue was embedded in Sakura Tissue Tek O.C.T. Compound and frozen in 2-Methylbutan cooled with liquid nitrogen. Tissues were cut at a Leica CM3050 S cryostat into 20 μm serial transverse sections at −20°C and subsequently stored at −80°C. Human cerebellar sections were incubated for 20 min in Polyscience L.A.B. solution for antigen retrieval at room temperature. Afterwards they were washed 3 times with PBS and blocked in 5% normal donkey serum in 0.3% PBS-T for 90 min. They were then incubated with the appropriate primary antibodies against PCP2 as PC marker and p-p53 (**Supplementary Table 2**) at 4°C overnight. The following day, after 6 washes of 10 min with PBS, 3 h incubation with secondary donkey antibodies coupled to Alexa488, Cy3 or Alexa647 (Jackson labs) diluted at 1:1000 in PBS-T were performed. After 6 times 10 min PBS washes, the slides were cover-slipped with glycerol:PBS (3:7) medium.

For immunostaining of murine cerebellum and spinal cords, mice were perfused with PBS and 4% PFA following 4% PFA post-fixation overnight at 4°C. On the following day, the brain and spinal cord were removed and washed in PBS. The cerebellum was then separated from the rest of the brain and split at the mid sagittal plane of the vermis. The hemispheres were removed, and half of the vermis was used for immunohistochemistry. The detailed protocol used for spinal cord dissection and identification of the lumbar L1 spinal segment has been previously reported^108^. The vermis or L1 segment was then embedded in warm 5% agar and serial transverse sections (vermis = 70 μm, spinal cord = 75 μm) were cut at the vibratome (Leica VT1000S). The sections were blocked with 5% normal donkey serum in PBS with 0.3% Triton X-100 (PBS-T; pH 7.4) for 90 min. Then the primary antibodies in blocking solution (**Supplementary Table 2**) were added overnight at room temperature. The next day, after the sections were washed 6 times for 10 minutes in PBS-T, 3 h incubation with secondary donkey antibodies coupled to Alexa488, Cy3 or Alexa647 (Jackson labs) diluted at 1:1000 in PBS-T were performed. Subsequently the sections were washed 6 times for 10 min in PBS and were mounted on slides with glycerol:PBS (3:7) medium.

For immunostaining of NMJs, mice were sacrificed or perfused and the muscle was dissected, immediately post-fixed with 4% PFA for 2 h and transferred into PBS. The next day, single muscle fibers were teased and washed 3 times in PBS for 10 min each followed by staining of the postsynaptic part of the NMJ with a-bungarotoxin (BTX) labeled with Alexa Fluor 555 in PBS for 20 min. Subsequently, the muscle fibers were washed 5 times in PBS for 10 min and blocked with 5% donkey serum in PBS with 0.3% Triton X-100 (PBS-T) for 1 h. Mouse anti-Neurofilament (NF) and anti-Synaptic vesicle 2 (SV2) antibodies to immunolabel the presynaptic aspect of the NMJ were applied in blocking solution overnight at 4°C (**Supplementary Table 2**). The muscle fibers were then washed 3 times for 10 min in PBS. Secondary antibodies were applied for 1 h in PBS-T at room temperature. Finally, the muscle fibers were washed 3 times in PBS for 10 min and mounted on slides covered with glycerol:PBS (3:7) as previously described^24^.

### Confocal imaging and analysis

For PC quantification and lobule-specific analysis, 70 μm vermis sections were scanned using a 10x objective (NA = 0.40). A z-step size of 4.0 μm was chosen to include PCP2^+^ PC somata. Leica LAS X software automatically stitched individual horizontal image planes into a full image of each vermis section. Each stack was split into two smaller stacks (~35 μm) for easier quantification of PCs and lobule sizes. The mean was calculated from each stack. Quantitative analysis of the vermis sections was done with Leica LAS AF software. The area of the entire vermis and individual lobules was measured using manual tracing. This method was also used to outline and quantify the molecular layer, PC layer, GC layer, and white matter for each of the 10 lobules. The perimeter of the PC layer was manually traced, and PCs were counted within each lobule. Three vermis sections per animal were analyzed.

For all synaptic density scans, a segment of the molecular layer from a single lobule was selected, and images were taken using a 63x oil objective (NA = 1.30) for optimal magnification. A z-step size of 0.4 μm was used to capture detailed images of synapses on PC somata and dendrites. The “z-compensation” function adjusted laser intensity at each z-step to ensure balanced signal quality while avoiding sample damage. Two scans were performed per animal. Synaptic density on PCs was analyzed using Leica LAS X software. For each investigated lobule, a minimum 5 PCs were randomly selected for analysis of VGLUT1^+^, VGLUT2^+^, and VGAT^+^ synapses. Synapses were manually counted on the soma and on dendritic segments at two distances (0–50 μm and 50–100 μm) from the soma. To minimize background-related errors, only synapses visible in at least two consecutive z-stack images were included.

### Intracellular recordings of Purkinje cells and granule cells in cerebellar sagittal slices

Whole-cell patch-clamp recordings from PCs and GCs were performed as previously described^109,110^ at P5 for PCs and P10 for both PCs and GCs. Following decapitation, the skull was opened, and the cerebellum was carefully extracted. Lateral portions of the cerebellum were trimmed to facilitate mounting in the vibratome chamber (HM 650 V, Microm, Thermo Fisher Scientific, UK), which was filled with ice-cold (~4°C), oxygenated (95% O_2_ / 5% CO_2_) sucrose-based artificial cerebrospinal fluid (aCSF) containing (in mM): 2.5 KCl, 1.1 CaCl_2_, 4 MgCl_2_, 25 NaHCO_3_, 1.25 NaH_2_PO_4_·H_2_O, 10 D-glucose, and 220 sucrose. Parasagittal cerebellar slices (300 μm thick) were prepared and transferred to a holding chamber containing standard aCSF, which was also used for recordings. This recording aCSF consisted of (in mM): 125 NaCl, 2.5 KCl, 1.25 NaH_2_PO_4_·H_2_O, 26 NaHCO_3_, 3 D-glucose, 1.1 CaCl_2_·H_2_O, 1 MgSO_4_·7H_2_O, and 17 sucrose (310 mOsm, pH 7.3, equilibrated with carbogen). Slices were incubated at 35°C for 30 minutes, then maintained at room temperature (21–23°C) until use. For recordings, individual slices were transferred to a recording chamber and continuously perfused with oxygenated aCSF at room temperature. PCs were visually identified by their position in the PC layer and their large soma, as well as their distinctive morphology featuring a prominent, expansive dendritic tree extending into the molecular layer. GCs were also visually identified by their location in the GC layer and their small soma, along with a minimal dendritic arbor. Patch pipettes were pulled from borosilicate glass (GB200F-10, Science Products, Hofheim am Taunus) using a Flaming-Brown puller (P-97, Sutter Instruments, CA) to resistances of ~4–7 MΩ for PCs and ~8–10 MΩ for GCs. Pipettes were filled with an intracellular solution containing (in mM): 150 K-D-gluconate, 10 NaCl, 10 HEPES, 3 Mg-ATP, 0.3 Na-GTP, and 0.05 EGTA, adjusted to pH 7.3 with KOH and to an osmolarity of 290–300 mOsm/kg H_2_O. Recordings were acquired in current-clamp or voltage-clamp mode using a HEKA EPC10/2 amplifier (HEKA Elektronik, Lambrecht/Pfalz, Germany) and digitized at 20 kHz with Patchmaster software (HEKA Elektronik). Signals were low-pass filtered at 3 kHz (DC–3 kHz; CyberAmp, Molecular Devices). RMP and spontaneous activity were recorded following a 10-second baseline with no current injection. Only cells with a RMP of −45 mV or lower, AP amplitudes of at least 30 mV, and the ability to repetitively fire 10 or more AP were included in further analysis as previously reported^111,112^. Passive membrane properties were assessed in current-clamp mode by holding the cells at −80 mV membrane potential to inhibit spontaneous activity and injecting brief (300 ms) alternating current steps: 4 pA increments for PCs and 1 pA for GCs. Frequency-current (F–I) relationships were determined by injecting 1-second depolarizing current steps (10 pA for PCs, 5 pA for GCs). Excitatory postsynaptic currents (EPSCs) were evoked by stimulation of parallel fibers for PCs or mossy fibers for GCs while holding the membrane potential at −70 mV. To isolate excitatory transmission during parallel fiber stimulation, 10 μM bicuculline was added to the bath solution to block GABA_A_ receptor-mediated inhibition. The stimulation pipette, filled with recording aCSF, was positioned in the molecular layer for parallel fiber or granular layer for mossy fiber simulation. The stimulus intensity was adjusted to elicit EPSCs with amplitudes between 200–600 pA to avoid action currents. Stimulation consisted of five pulses at 200 Hz for parallel fibers or 100 Hz for mossy fibers, with a duration of 200 μs per pulse. Data were analyzed offline using Patchmaster software (HEKA Elektronik).

### USV analysis

USV experiments were performed at the Mouse NeuroBehavior Core of Columbia University. To elicit USV emission, neonatal mouse pups were gently separated from the dam and placed in a small plastic container lined with a 0.5 cm layer of fresh bedding. The cage lid was immediately and gently placed back, to avoid agitating the dam and the remaining pups in the nest. The container with the isolated pup was immediately placed inside a sound-attenuating chamber (Med Associates) to minimize background noise and environmental disturbances. USVs were recorded using an ultrasound-sensitive microphone (Avisoft UltraSoundGate condenser microphone CM16, Avisoft Bioacoustics; frequency range: 10–180 kHz) connected to a PC running Avisoft Recorder software. After each 3-minute recording session, the pup was marked to prevent repeated handling on the same day and returned to the nest. This procedure was repeated until all pups in the litter were tested. Recordings were performed on postnatal days P3, P5, P7 and P9. USV recordings were analyzed using Avisoft SASLab Pro software. Only frequencies above 35 kHz were analyzed. Spectrograms were generated using a Fast Fourier Transform (FFT) with a 512-length, frame size 100%, 50% overlap, and a Hamming window. Calls were manually labeled and parameters such as call number, duration, peak frequency, and inter-call interval were extracted for statistical analysis. In line with the Reduction principle of the 3Rs Guidelines in animal research, we minimized the number of animals used for USV analysis by reusing data from control and SMA mice across Fig. 4, Fig. 7, and Fig. S9. These groups were shared across experiments to reduce the overall number of animals without compromising scientific integrity. The AAV9-L7-6-SMN–treated mutants were littermates of the other groups, and all recordings were performed concurrently.

### Statistics

In this manuscript, N refers to the number of patients or mice in each group, and n refers to the number of cells analyzed. Results are presented as the mean ± standard error (SE), based on at least three independent experiments involving three or more animals or patients per experimental group, unless specified otherwise in the figure legend. To assess the symmetry of data distribution, the Shapiro-Wilk normality test was applied. For comparisons between two groups, statistical analysis depended on the data’s distribution and the equality of standard deviations (SDs). If the data followed a parametric distribution and the SDs were equal, an unpaired t-test or multiple t-test were used. When the SDs were unequal, Welch’s t-test was applied. For nonparametric distributions, Mann-Whitney or multiple Mann-Whitney tests were performed. For comparisons among three groups or more, a one-way ANOVA followed by Tukey’s multiple comparison test was used for parametric distributions. Nonparametric distributions were analyzed using the Kruskal-Wallis test with Dunn’s correction. In cases where SDs were unequal, a Welch’s ANOVA with Dunnett’s T3 multiple comparison test was performed. For time-course comparisons involving more than two groups, a two-way ANOVA with Tukey’s multiple comparison test was applied. The Log-rank (Mantel–Cox) test was used for survival analysis. The statistical tests used for each experiment are indicated in the respective figure legends. All statistical analyses were conducted using GraphPad Prism 10, and p-values are reported within the figures.

## Supplementary Material

Supplementary Files

This is a list of supplementary files associated with this preprint. Click to download.

- GerstneretalCerebellumSMASUPPLEMENTARYfinal.docx

## Highlights

- Type I SMA patients and severe SMA mice exhibit selective p53-dependent death of PCs
- Synaptic loss and circuit dysfunction exacerbate cerebellar pathology in SMA mice
- SMA mice display social communication deficits during early postnatal development
- Cerebellar pathology contributes to motor and communication deficits in SMA mice
